# A deep learning framework for ^18^F-FDG PET imaging diagnosis in pediatric patients with temporal lobe epilepsy

**DOI:** 10.1007/s00259-020-05108-y

**Published:** 2021-01-09

**Authors:** Qinming Zhang, Yi Liao, Xiawan Wang, Teng Zhang, Jianhua Feng, Jianing Deng, Kexin Shi, Lin Chen, Liu Feng, Mindi Ma, Le Xue, Haifeng Hou, Xiaofeng Dou, Congcong Yu, Lei Ren, Yao Ding, Yufei Chen, Shuang Wu, Zexin Chen, Hong Zhang, Cheng Zhuo, Mei Tian

**Affiliations:** 1 Department of Nuclear Medicine and PET-CT Center, The Second Hospital of Zhejiang University School of Medicine, Hangzhou, Zhejiang China; 2 College of Information Science & Electronic Engineering, Zhejiang University, Hangzhou, Zhejiang China; 3 Key Laboratory for Biomedical Engineering of Ministry of Education, Zhejiang University, Hangzhou, Zhejiang China; 4 Department of Pediatrics, The Second Hospital of Zhejiang University School of Medicine, Hangzhou, Zhejiang China; 5 Department of Neurology, Epilepsy Center, The Second Hospital of Zhejiang University School of Medicine, Hangzhou, Zhejiang China; 6 Center of Clinical Epidemiology & Biostatistics, The Second Hospital of Zhejiang University School of Medicine, Hangzhou, Zhejiang China; 7 College of Biomedical Engineering and Instrument Science, Zhejiang University, Hangzhou, Zhejiang China

**Keywords:** Deep learning, Epilepsy, Pediatrics, Positron emission tomography (PET), Glucose metabolism

## Abstract

**Purpose:**

Epilepsy is one of the most disabling neurological disorders, which affects all age groups and often results in severe consequences. Since misdiagnoses are common, many pediatric patients fail to receive the correct treatment. Recently, ^18^F-fluorodeoxyglucose positron emission tomography (^18^F-FDG PET) imaging has been used for the evaluation of pediatric epilepsy. However, the epileptic focus is very difficult to be identified by visual assessment since it may present either hypo- or hyper-metabolic abnormality with unclear boundary. This study aimed to develop a novel symmetricity-driven deep learning framework of PET imaging for the identification of epileptic foci in pediatric patients with temporal lobe epilepsy (TLE).

**Methods:**

We retrospectively included 201 pediatric patients with TLE and 24 age-matched controls who underwent ^18^F-FDG PET-CT studies. ^18^F-FDG PET images were quantitatively investigated using 386 symmetricity features, and a pair-of-cube (PoC)-based Siamese convolutional neural network (CNN) was proposed for precise localization of epileptic focus, and then metabolic abnormality level of the predicted focus was calculated automatically by asymmetric index (AI). Performances of the proposed framework were compared with visual assessment, statistical parametric mapping (SPM) software, and Jensen-Shannon divergence-based logistic regression (JS-LR) analysis.

**Results:**

The proposed deep learning framework could detect the epileptic foci accurately with the dice coefficient of 0.51, which was significantly higher than that of SPM (0.24, *P* < 0.01) and significantly (or marginally) higher than that of visual assessment (0.31–0.44, *P* = 0.005–0.27). The area under the curve (AUC) of the PoC classification was higher than that of the JS-LR (0.93 vs. 0.72). The metabolic level detection accuracy of the proposed method was significantly higher than that of visual assessment blinded or unblinded to clinical information (90% vs. 56% or 68%, *P* < 0.01).

**Conclusion:**

The proposed deep learning framework for ^18^F-FDG PET imaging could identify epileptic foci accurately and efficiently, which might be applied as a computer-assisted approach for the future diagnosis of epilepsy patients.

**Trial registration:**

NCT04169581. Registered November 13, 2019

Public site: https://clinicaltrials.gov/ct2/show/NCT04169581

**Supplementary Information:**

The online version contains supplementary material available at 10.1007/s00259-020-05108-y.

## Introduction

Epilepsy is one of the most common serious pediatric neurological disorders with a prevalence of 0.5–1% [1, 2]. The temporal lobe epilepsy (TLE) is the most frequent subtype of focal epilepsy, and 80–90% TLE patients show hypometabolism on ^18^F-fluorodeoxyglucose positron emission tomography (^18^F-FDG PET) imaging at the interictal state [3, 4]. ^18^F-FDG PET has played an important role in epileptic patient management, since it has higher detection sensitivity (86% vs. 73–80%) than those of electroencephalogram (EEG), single-photon emission computed tomography (SPECT), or magnetic resonance imaging (MRI) [5–9]. In the clinical setting, PET image analysis should be done by drawing region-of-interest (ROI) or volume-of-interest (VOI) and then calculating the standard uptake value (SUV), SUV ratio (SUVR), or asymmetric index (AI) [10–12]. However, this analysis method might lead to significant diagnostic bias, since it is highly depended on the physician`s own experience [13]; therefore, computer-aided diagnosis approach is warranted.

Statistical parametric mapping (SPM) is so far the most widely used computer-aided approach for PET imaging analysis [11, 12], in which all subjects need to be normalized into the same standard space, followed by performing voxel-wise statistical comparison between patients and normal controls. Previous ^18^F-FDG PET studies have demonstrated that SPM analysis could improve the localization of epileptic focus [14, 15]. However, it only makes use of a small portion of image information, and its voxel-based morphometry (VBM) method tend to yield false positives [16] especially when comparing pediatric data with adult healthy controls due to imperfect registration [13]. Recently, radiomics analysis attracted widespread attentions, since it can unravel the hidden information in digital images and potentially improve diagnostic, prognostic, predictive, and classification accuracy [17]. Nonetheless, radiomics analyses are commonly limited to segmentation and localization. Thus, it is crucial to develop an accurate and efficient method for epileptic focus identification in pediatric patients.

Although many studies demonstrated that deep learning method could achieve comparable or even better results than visual assessment by experienced physicians in diagnosis of various diseases including lung cancer, Alzheimer’s disease, and Schizophrenia [18–20], to the best of our knowledge, no publication has been found on deep learning-assisted identification of epileptic focus using ^18^F-FDG PET imaging. We consider that there are three major challenges for developing a deep learning-based ^18^F-FDG PET diagnosis approach for pediatric epilepsy: (1) it is very difficult to localize the epileptic focus which might present as either hypo- or hypermetabolism; (2) it is pretty difficult to get the accurate label for training of deep learning since the boundary of epileptic focus is too intricate to be delineated explicitly; (3) it is difficult to include normal pediatric controls who have done ^18^F-FDG PET scans due to the ethical concern. To overcome the above-mentioned challenges, we hypothesize that the epilepsy is strongly correlated to the high-dimensional interhemispheric symmetricity changes in PET images. We used radiomics features with symmetric information to diagnose TLE and confirm this hypothesis. Then we proposed a novel deep learning method based on a Siamese convolutional neural network (CNN) to track the metabolic symmetricity of ^18^F-FDG PET images for the detection of epileptic focus.

## Materials and methods

### Subjects

We retrospectively reviewed a dataset of 201 pediatric patients with TLE (92 girls and 109 boys, age 11.19 ± 3.43 years) who underwent ^18^F-FDG PET-CT, EEG, and MRI from November 2013 to April 2019. Exclusion criteria were the following: (1) poor image quality, e.g., severe image artifacts due to head movement, and (2) negative finding on ^18^F-FDG PET scan, (3) incomplete clinical data, and (4) missing EEG or MRI data. To determine a negative finding (no significant abnormal finding) on ^18^F-FDG PET scan, all images were reviewed by the team of experts to reach a consensus. A total of 136 cases were included for the following analysis (including 57 girls and 79 boys with ages of 10.84 ± 3.35 years).

An age-matched control group dataset (6 girls and 18 boys, age 11.58 ± 2.83 years) was built by retrospectively reviewing patients with extracranial lymphoma who had no history of neurologic disorders, psychiatric illnesses, chemotherapy, or radiotherapy. This retrospective study was approved by the Human Subject Research Ethics Committee of The Second Hospital of Zhejiang University School of Medicine, and the requirement of informed consents was waived (Approval Number: 2019162).

### ^18^F-FDG PET imaging acquisition and visual assessment

All subjects were injected with 3.7 MBq/Kg ^18^F-FDG after fasting at least 6 h and then rested with eyes closed in dark and quiet environment for a 40-min uptake period. PET-CT brain images were acquired on a PET-CT scanner (Biograph mCT; Siemens Medical Solutions), using a 5-min bed position and 3D whole-head acquisition. Data were reconstructed by a vendor-provided software using Fourier recombination (FORE) and attenuation-weighted ordered-subsets expectation maximization (AW-OSEM). The reconstructed resolution of PET images was almost isotropic with a full width at half maximum (FWHM) of 4.4 mm in the center and a FWHM of 4.8 mm at 10 cm off-axis. Images were then resliced into 2-mm-thick slices and reoriented to the coronal-transaxial-sagittal orientation on Siemens medical workstation.

^18^F-FDG PET images were visually assessed by two physicians specialized in epilepsy diagnosis (YL, XW) who were unblinded to the seizure semiology, EEG, and MRI findings. All epileptic foci were manually delineated using ITK-SNAP software (version 3.6.0; https://www.itksnap.org). For inconsistent cases, the final decision was made by the team of experts to reach a consensus through cross-review.

### ^18^F-FDG PET image preprocessing and dataset separation

The ^18^F-FDG PET images were spatially normalized to an in-house symmetric template by the symmetric diffeomorphic registration using Advanced Normalization Tools (version 2.1; https://stnava.github.io/ANTs) [21]. All warped images were then transformed to Z-maps using Fisher’s Z-transformation. After that, PET images were randomly divided into two sets: training (106 patients and 9 controls) or testing (30 patients and 15 controls) set. The training set was involved in the model training for both radiomics analysis and deep learning, while the testing set was used to evaluate the performance of well-trained models.

### Radiomics-based ^18^F-FDG PET image analysis

The radiomics-based ^18^F-FDG PET analysis consisted of feature extraction, feature selection, and model training. In the feature extraction, the symmetricity features of the bilateral temporal lobes were quantitatively investigated to imitate the visual assessment that bilateral glucose uptake was compared simultaneously. The radiomics features (totally 386 dimensions, described in Supplementary Materials), including 344 multiscale wavelet features, 36 texture features, and 6 intensity features, were extracted from the bilateral temporal lobes. The multiscale wavelet features included intensities and textures from Coiflet 1 wavelet (coif1) transformed images. The texture features were calculated based on histogram, gray-level co-occurrence matrix (GLCM), neighborhood gray-tone difference matrix (NGTDM), and gray-level zone size matrix (GLZSM). The intensity features included mean, variance, skewness, kurtosis, energy, and entropy. The symmetricity feature was obtained by calculating the absolute distance between the radiomics features of the left and right lobes according to the following equation:

$$ {d}_i=\left|{\boldsymbol{f}}_i^{left}-{\boldsymbol{f}}_i^{right}\right|\kern0.5em \forall i=1,2,\cdots, 386 $$

where ${f}_i^{\mathrm{left}}$ and ${f}_i^{\mathrm{right}}$ represent the number *i* feature of either the left or right lobe. The symmetricity vector *d* = (*d*_1_, *d*_2_, ⋯, *d*_386_) was then built to represent the individual inter-hemispheric temporal lobe symmetricity and formed a symmetricity distance matrix. Column-wise Z-transformation was applied to the symmetricity distance matrix to obtain the normalized symmetricity matrix.

For feature selection, the hierarchical clustering (HC)—a widely used clustering method—was applied to the normalized symmetricity matrix to obtain *k* clusters (*k*represents the number of clusters). In each cluster, the most significant symmetricity feature was selected by maximum absolute Fisher inter-intra class variance ratio (FICVR) [22], which can be defined in the following equation:

$$ \mathrm{FICVR}=\left|\raisebox{1ex}{$\left({\mu}_p-{\mu}_c\right)$}\!\left/ \!\raisebox{-1ex}{$\sqrt{\sigma_p^2+{\sigma}_c^2}$}\right.\right| $$

where *μ*_*p*_ stands for the mean symmetricity features of the patient and *μ*_*c*_ represents that of the control, while *σ*_*p*_ and *σ*_*c*_ denote the standard deviations of the patient and the control, respectively. The FICVR of a feature represents its capability of distinguishing the patient from the control.

To verify the advantages of our selected features, this study evaluated the classification performances of our selected features, low-order features (intensity features), mono-features (features with favorable classification metrics), and multi-features (all features and principal component analysis (PCA) selected features) based on logistic regression models. The classification metrics such as accuracy, sensitivity, specificity, and receiver operating characteristic (ROC) were displayed. To improve the robustness of the logistic regression models, the training set was divided into 10 groups through a 10-fold cross-validation procedure, which was consisted of 10 iterations: in each iteration, one group was used as the validation set, while the rest of the training sets were used for model training [23]. This procedure was repeated for 10 times to obtain the average performance.

### Deep learning-based epileptic focus localization

**Fig. 1:**
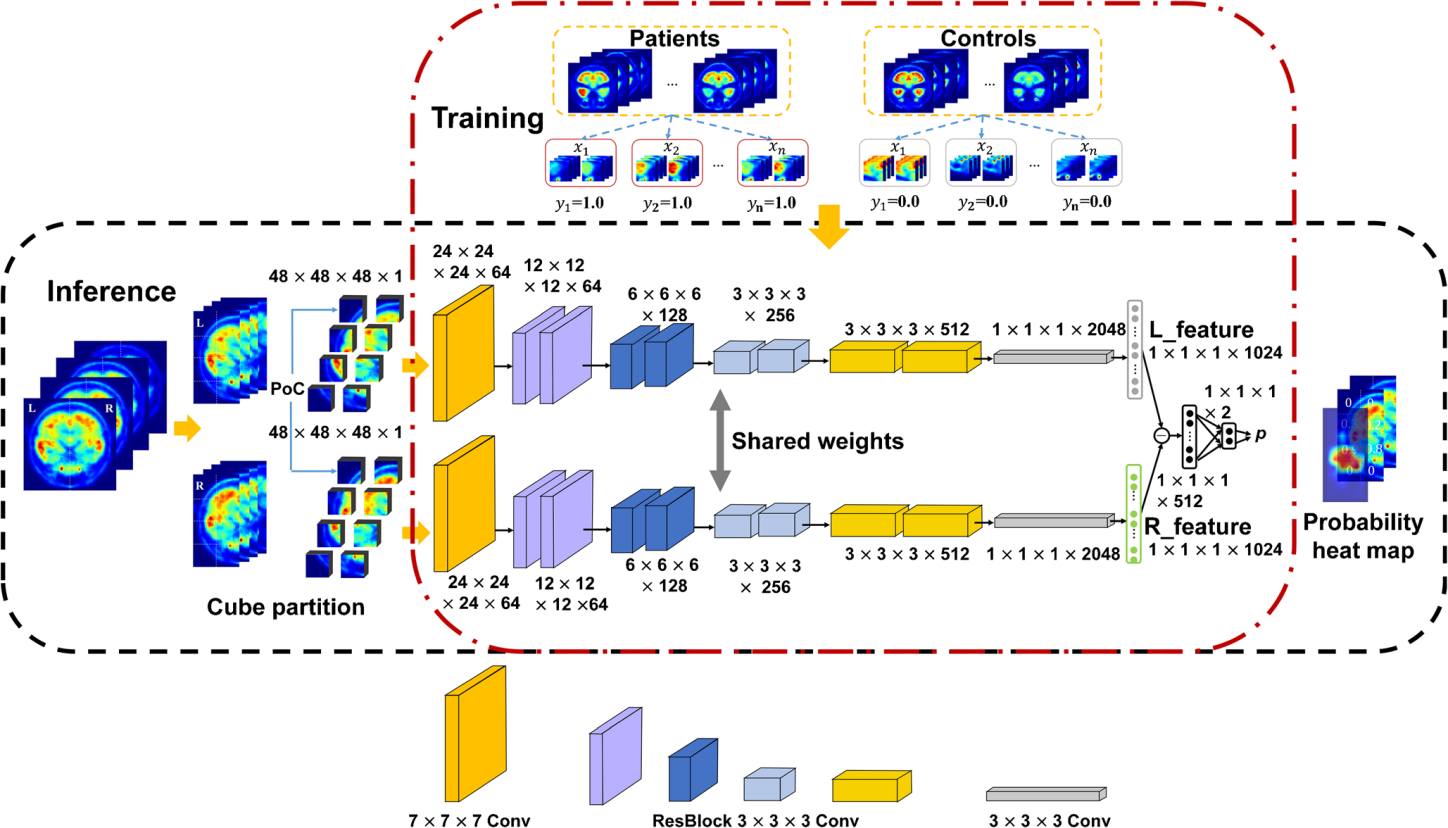
The proposed framework for epileptic focus detection

A deep learning approach, symmetricity-driven Siamese CNN, was proposed to localize the epileptic focus. This proposed framework, which consisted of two identical 18-layer residual convolution neural networks (ResNet) [24], was firstly used to extract deep features of bilateral image cubes automatically and then to localize the focus by calculating the feature differences (Fig. 1). The procedures of training and inference were demonstrated in the red and black dashed-dotted boxes, respectively. In the procedure of training, the input *x* belong to the sample image set *X*; while the corresponding outpu *y* belong to the label set *Y*. It is noted that *y* = 0.0 and *y* = 1.0 represent the normal and abnormal image cubes, respectively. Therefore, the epileptic focus detection could be considered as a symmetricity-driven binary classification. In the procedure of inference, the output probability *p* of the framework was obtained on testing set. The above-mentioned procedures were implemented by using PyTorch (version 1.0; https://pytorch.org).

To obtain the inputs of Siamese CNN, the right and left parts of PET images were partitioned to pairs-of-cubes (PoCs), and each PoC was consisted of *n* × *n* × *n* pixels (here we set *n* = 48). Then the positive (with focus) and negative (without focus) PoCs were obtained from the patients and controls. To balance the sample sizes of positive and negative PoCs, data augmentation was conducted on the training set, such as flipping, radial distortion, and intensity modification. In addition, sample weighting was used by setting relatively larger weights for minority samples in each training batch. The PoC-based training strategy could overcome the challenge of small training sample size in PET imaging and improve the framework robustness.

In the process of Siamese CNN training, PoCs were respectively fed to the two sub-networks to obtain two 1024-entry feature vectors. The absolute difference between the two feature vectors was then processed by a fully connected layer to get the prediction score. For parameters setting, we applied the standard stochastic gradient descent with learning rate of 0.01 and set weight decay as 0.005, batch size as 128, and training epoch as 16. In each training epoch, the trained model obtained the validation results on the validation set. The model with the best validation result was selected as the final model for the inference on the testing set.

In the procedure of inference for focus detection, the input PET images were partitioned into cubes to generate PoCs. The aforementioned procedure was executed on each PoC to obtain the output *p*. Eventually, a probability heat map for predicting the abnormal focus was achieved in one PET image, as illustrated in the last stage of Fig. 1. To evaluate the performance of our proposed method on PoC classification, this study calculated the Jensen-Shannon (JS) divergence of PoCs and applied a logistic regression (LR) classifier to discriminate normality and abnormality for comparison.

After detecting abnormal focus, the framework further calculated the SUVR and determined hyper- or hypometabolism by computing asymmetric index (AI):

$$ \mathrm{AI}=\frac{2\times \left(\mathrm{SUVR}\left(\mathrm{ipsilateral}\right)\hbox{-} \mathrm{SUVR}\left(\mathrm{contralateral}\right)\right)}{\mathrm{SUVR}\left(\mathrm{ipsilateral}\right)+\mathrm{SUVR}\left(\mathrm{contralateral}\right)} $$

If absolute AI was larger than the threshold (e.g., 0.15) for three consecutive slices, the focus was determined as severe metabolic abnormality, otherwise mild. The hypo- or hypermetabolism was determined by the sign of AI, i.e., negative for hypo- and positive for hyper.

### Statistical analysis

To evaluate the accuracy and consistency of the proposed method, results were compared with those of SPM (version 8; http://www.fil.ion.ucl.ac.uk/spm/) and physicians with different experience levels, i.e., physicians specialized in epilepsy diagnosis (specialist), junior nuclear medicine physicians (junior), senior nuclear medicine physicians (senior), and a senior neurologist (neurologist). In SPM analysis, all PET images were spatially normalized to standard stereotactic space by an in-house symmetric ^18^F-FDG PET template, followed by smoothing with 8-mm FWHM Gaussian kernel. Two-tailed inference and cluster size level of 100 were used as prior study [25]. SPM results were classified into four levels according to the uncorrected *P* values, i.e., normal (*P* > 0.05), probably normal (0.01 < *P* ≤ 0.05), probably abnormal (0.001 ≤ *P* < 0.01), and abnormal (*P* < 0.001).

The diagnosis and localization results of proposed method were compared with those of SPM analysis and physicians with different levels. The physicians decided each case as normal, probably normal, probably abnormal, or abnormal and then manually delineated the epileptic foci for all suspected “abnormal” cases. Dice coefficient [26] was used to quantify the similarity of proposed method, physicians, and SPM results to the reference standard. Wilcoxon signed-rank test was employed to compare the dice coefficients of different methods. The percentage of correct diagnosis, ROC curves, sensitivity, and specificity were reported.

The glucose metabolic levels were classified into four categories: severe hypometabolism, mild hypometabolism, mild hypermetabolism, or severe hypermetabolism. The metabolic abnormality level determination results were compared between proposed method and physicians. Physicians first found the epileptic foci and estimated the metabolic abnormality levels of the foci blinded to clinical information including seizure semiology, EEG, and MRI findings. After that, physicians utilized the clinical information to re-evaluate foci and metabolic levels. At last, we compared the physician diagnosis results blinded or unblinded to the clinical information and those of the proposed framework. Wilcoxon signed-rank test was employed to evaluate physician determination of hypo- and hypermetabolism blinded or unblinded to clinical information. McNemar’s test was employed to compare physician visual assessments and proposed method. *P* value less than 0.05 (*P* < 0.05) was considered statistically significant.

## Results

### Radiomics-based epilepsy diagnosis

**Fig. 2:**
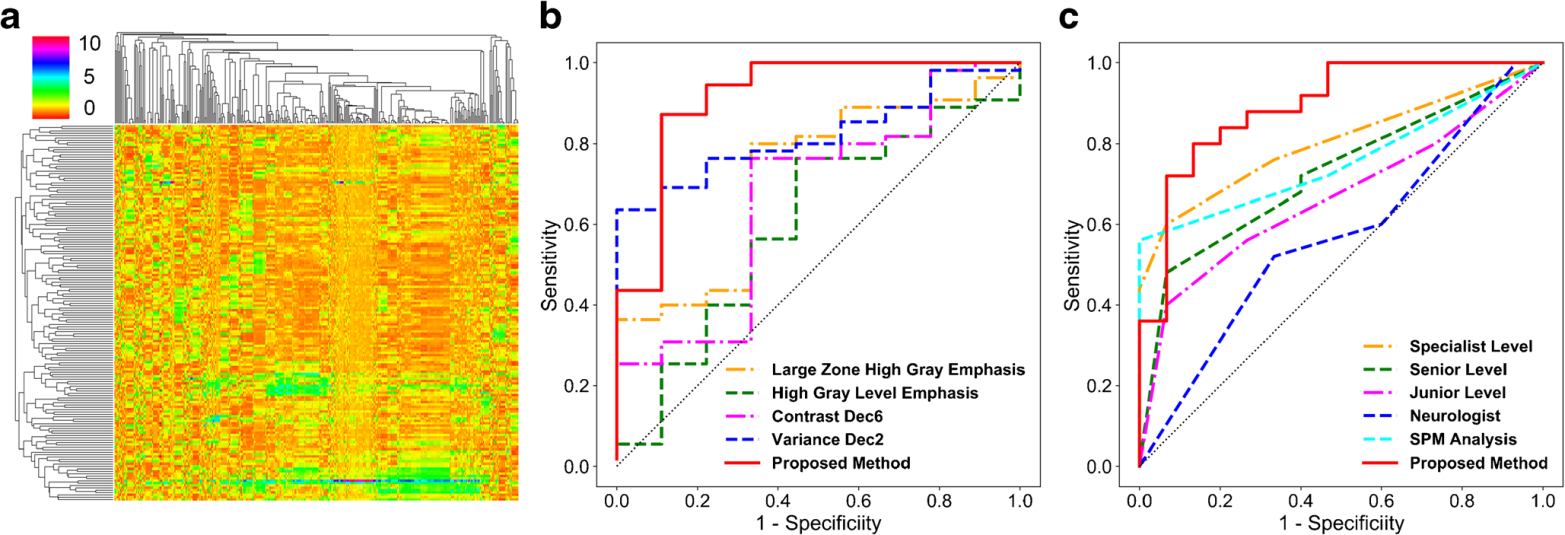
**a** Symmetricity feature heat map; **b** ROC curves of mono- and our selected features; and **c** ROC curves of visual assessment, SPM analysis, and the proposed method

The heat map of symmetricity features was demonstrated in Fig. 2a. The rows represented images of the control and the patient, and columns represented symmetricity features. The colors of the heat map varied from orange to purple, which represented the values of symmetricity features changed from small to large. All symmetricity features were grouped into 10 clusters by HC, from which the feature with maximum FICVR was selected (totally 10 features were selected, see Table S1 in Supplementary Materials). As shown in Fig. 2b, the ROC curve of our proposed method based on 10 selected features outperformed those classification methods using 4 single features in mono-feature category with largest area under curves (AUCs). Table 1 illustrated all classification metrics of low-order, mono-, multi-, and our selected features. The “high gray-level zone emphasis” feature had the highest sensitivity (0.82); the proposed classification method had the highest AUC (0.92), accuracy (0.81), and specificity (0.89), which suggested the proposed cluster-based feature selection method could purposely select the most TLE-correlated symmetricity features.

**Table 1:** AUC, accuracy, sensitivity, and specificity of low-order, mono-, multi-, and our selected features

Feature category		AUC	Accuracy	Sensitivity	Specificity
Low-order features	Mean	0.65	0.50	0.47	0.67
	Variance	0.59	0.53	0.53	0.56
Mono-feature	LZHGE	0.72	0.53	0.51	0.67
	HGLZE	0.60	0.73	0.82	0.42
	Contrast dec6	0.67	0.69	0.69	0.67
	Variance dec2	0.82	0.77	0.76	0.78
Multi-feature	All features	0.76	0.59	0.58	0.67
	PCA selected features	0.67	0.64	0.69	0.33
Our proposed	10 selected features	0.92	0.81	0.80	0.89

*AUC* area under the curve, *LZHGE* large zone high gray emphasis, *HGLZE* high gray-level zone emphasis, *PCA* principal component analysis

The diagnosis results of the proposed method, the SPM analysis, and visual assessments were shown in Table 2 and Fig. 2c. The experienced physicians had relative higher AUC and accuracy than the ones with less experience in epilepsy diagnosis. The SPM analysis had similar AUC and accuracy to the specialists (AUC 0.76 vs. 0.80; Accuracy 0.73 vs. 0.75). The proposed radiomics-based analysis had the highest AUC (0.89), accuracy (0.82), sensitivity (0.84), and specificity (0.80) than the SPM analysis and visual assessments, suggesting a strong correlation between high-dimensional symmetricity features and epilepsy. This finding supported our hypothesis that epilepsy had intrinsic high-dimensional metabolic asymmetricities that cannot be easily recognized by visual assessment.

**Table 2:** AUC, accuracy, sensitivity, and specificity of epilepsy diagnosis among the physicians with different experiences, SPM analysis, and the proposed method

Group	AUC	Accuracy	Sensitivity	Specificity
Specialized level	0.80	0.75	0.64	0.93
Senior level	0.73	0.65	0.48	0.93
Junior level	0.67	0.64	0.60	0.70
Neurologist	0.57	0.58	0.53	0.67
SPM analysis	0.76	0.73	0.56	1.00
Proposed method	0.89	0.82	0.84	0.80

*AUC* area under the curve, *SPM* statistical parametric mapping

### Deep learning-based epileptic focus localization

**Fig. 3:**
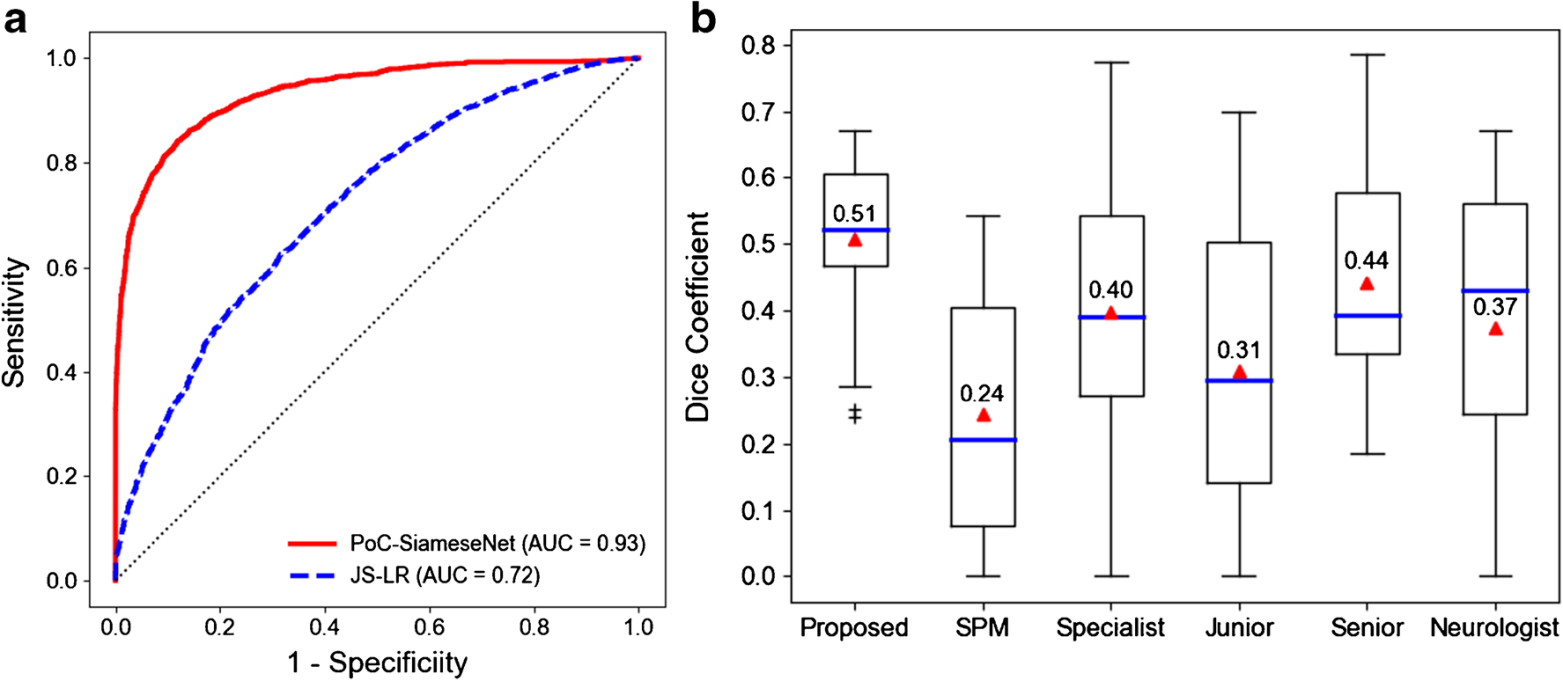
**a** ROC curves of JS-LR and PoC-Siamese network; **b** dice coefficients obtained by the physicians with different levels, SPM analysis, and the proposed method

**Fig. 4:**
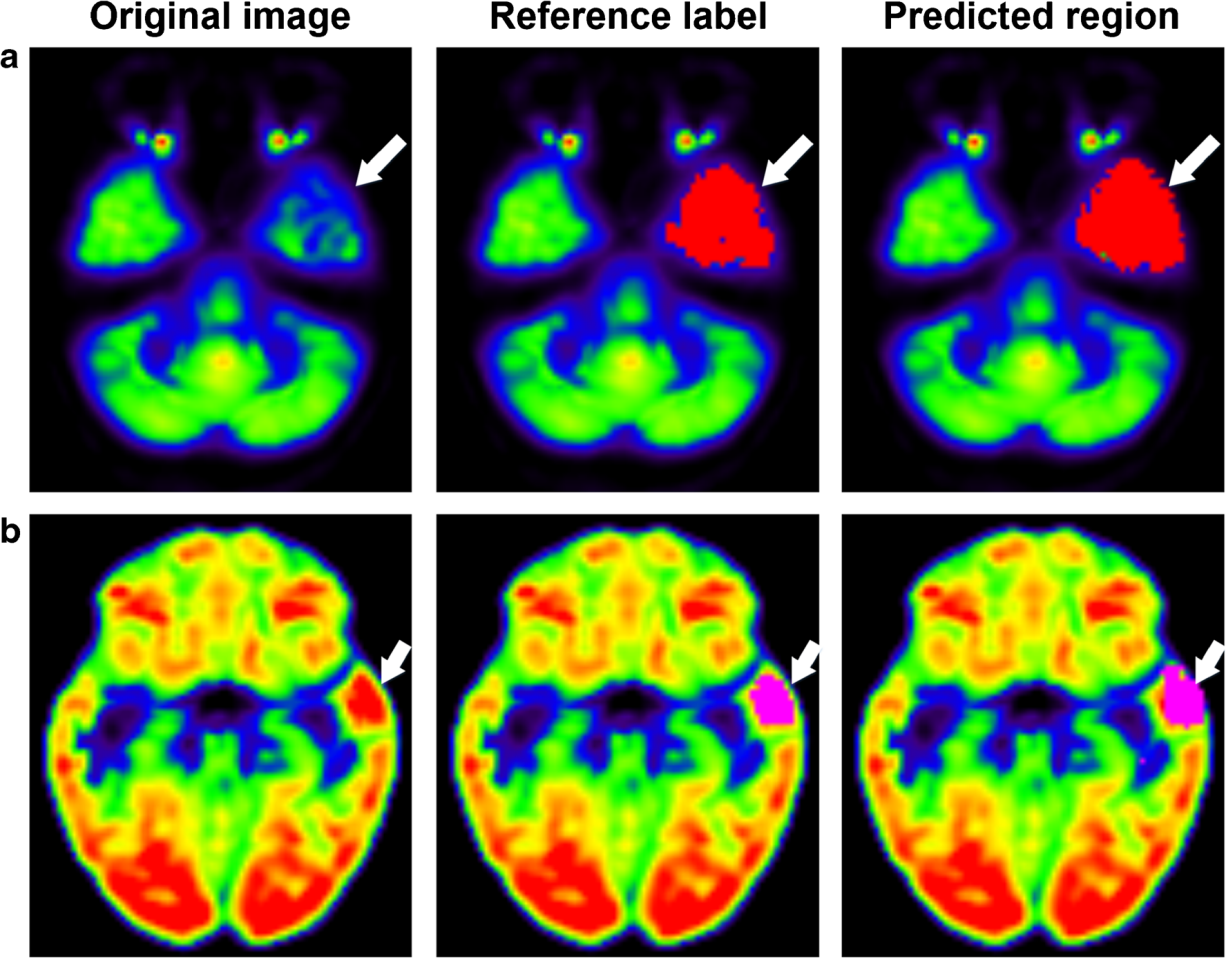
Two examples of the proposed epileptic focus localization of **a** hypometabolism and **b** hypermetabolism

A total of 51,689 PoCs were extracted from 106 patients and 9 controls, in which 60% of PoCs were used for training, 20% of PoCs for validation, and 20% for testing. The proposed method could accurately detect abnormal PoCs (AUC = 0.93), which is better than the JS-LR (AUC = 0.72) (Fig. 3a). As shown in Fig. 3b, the results demonstrated that the proposed approach had the highest average dice coefficient (0.51) compared with the physicians (0.31–0.44) and SPM analysis (0.24). Wilcoxon signed-rank test showed that proposed method significantly outperformed SPM analysis (*P* < 0.01), junior physicians (*P* = 0.005–0.017), and neurologist (*P* = 0.038). The proposed method had a marginally significantly higher dice coefficient than senior physician (*P* = 0.068) and specialists (*P* = 0.09–0.17). Two examples of hypo- and hypermetabolism were presented in Fig. 4. Both hypo- and hypermetabolism regions predicted by the proposed deep learning method were consistent with the reference label. Moreover, the proposed method tended to be more aggressive by including slightly larger regions than the reference label to avoid false negatives (i.e., missed diagnosis). Such aggressiveness can be fine-tuned by a threshold parameter used in the framework.

### Determination of metabolic abnormality level

**Fig. 5:**
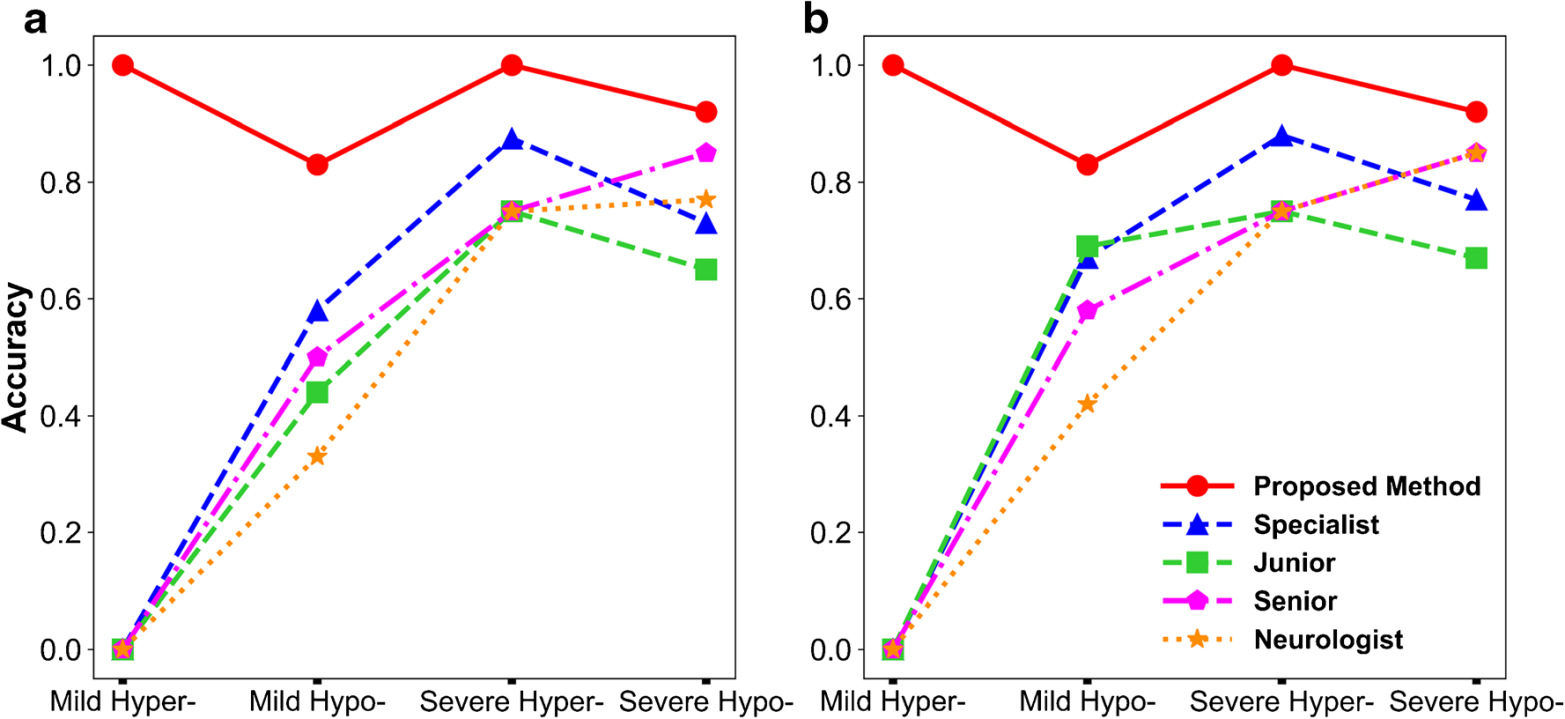
Comparison of metabolic abnormality level determination accuracy between proposed method and physicians blinded to clinical information (**a**) and unblinded (**b**)

The determination ability of metabolic abnormality level was compared between the proposed method and the physicians (see Fig. 5). All physicians failed to recognize mild hypermetabolism with or without access to the clinical information. All physicians (except for one junior nuclear medicine physician) had higher determination accuracy in terms of severe hypometabolism than in the mild ones (71% vs. 55%; *P* = 0.032). Consulting clinical information can significantly increase the determination accuracy of physicians for mild hypometabolism from 46 to 64% (*P* < 0.01). However, the impact of clinical information for severe hypermetabolism determination is very limited (Accuracy = 78% for both blinded and unblinded).

The proposed method had higher determination accuracy for both severe and mild abnormalities (94% and 85%, respectively) than physicians blinded (68% and 42%) to the clinical information or unblinded (75% and 59%), as presented in Table 3. The McNemar’s test showed that the proposed method significantly outperformed the physicians blinded or unblinded to clinical information (90% vs. 56% and 68%; *P* < 0.01). In particular, the proposed method successfully determined all the hypermetabolism (100%), which was superior to all the physicians.

**Table 3:** Accuracy of metabolic abnormality level determination between the physicians and the proposed method

Group	Severe hypometabolism (*n* = 13)	Severe hypermetabolism (*n* = 4)	Severe abnormality (*n* = 17)	Mild hypometabolism (*n* = 12)	Mild hypermetabolism (*n* = 1)	Mild abnormality(*n* = 13)
Physicians (blinded)	0.65	0.78	0.68	0.46	0.00	0.42
Physicians (unblinded)	0.74	0.78	0.75	0.64	0.00	0.59
Proposed method	0.92	1.00	0.94	0.83	1.00	0.85

## Discussion

This study proposed a deep learning method using a symmetricity-driven Siamese CNN for epilepsy diagnosis. The proposed method extracted high-dimensional symmetricity features of metabolism in epilepsy to detect both hypo- and hypermetabolism. Pair-of-cube training strategy was employed to learn metabolic symmetricity with limited imbalanced dataset. To the best of our knowledge, this is the first deep learning study to investigate epilepsy using ^18^F-FDG PET imaging.

The proposed deep learning framework can efficiently localize epileptic foci beyond visual assessment and conventional SPM analysis. Visual assessment is largely depended on the clinical experience of physicians [13]. Therefore, visual assessment varied between physicians and had low diagnosis accuracy of mild metabolic abnormality [25]. Conventional VBM-based SPM analysis could improve diagnostic accuracy, but it might be susceptible to high false positive rates [16] and only made use of a small portion of image information. In contrast, the proposed method can learn high-dimensional symmetricity features of epilepsy though Siamese CNN and achieve high diagnosis accuracies of both severe (94%) and mild metabolic abnormality (85%), confirming the hypothesis that epilepsy is closely related to high-dimensional metabolic symmetricity features.

Our method employed PoC training strategy to analyze metabolic symmetricity of epilepsy with limited and imbalanced pediatric ^18^F-FDG PET dataset. This strategy imitated the visual assessment that bilateral glucose uptake was compared simultaneously [27]. On the other hand, the pediatric PET dataset was usually very limited and imbalanced with much less controls [28]; conventional training method could thus fail to reach convergence or result in overfitting. The PoC training strategy used pairs of image cubes rather than whole images for data augmentation. Then, the PoCs were classified as positive or negative samples; such binary classification strategy can have better tolerance of the human-induced inaccuracies and bias when delineating epileptic boundaries. The regions localized by the proposed approach matched the best with the reference standard compared with all physicians and SPM analysis, which demonstrated the proposed PoC approach is helpful for boosting the performance.

The proposed symmetricity radiomics analysis model outperformed both visual evaluation and SPM analysis in diagnosis. This indicated there may exist complex metabolic alternations in both ipsilateral and contralateral temporal lobes. This finding was consistent with the most recent works that treated epilepsy as a whole-brain network-level disease [29, 30]. Moreover, previous studies also found that unilateral TLE may cause contralateral compensation mechanism, including contralateral structural alternations [31], increased functional connectivity [32], and hypermetabolism [33]. The contralateral compensation mechanism may further highlight symmetricity features to improve localization accuracy in turn. Notably, “high gray-level zone emphasis” (HGZE), one of the critical features found by the proposed method to describe the distribution of high gray-level zones, had the highest sensitivity. The results suggested more attention should be placed on temporal subregions that had relatively higher glucose uptake levels.

Machine learning approaches have been proposed to investigate epilepsy using other image modalities. T1-MRI radiomics analyses found abnormal texture features in hippocampus of mesial TLE, the right thalamus in juvenile myoclonic epilepsy [34], and the altered wavelet features in epileptic foci [35]. Support vector machine (SVM) combined with voxel-based morphometry (VBM) was proposed to distinguish presurgical mesial TLE from healthy controls using T1-MRI, fluid-attenuated inversion recovery (FLAIR), and/or diffusion tensor imaging (DTI) features [36–38]. These studies were limited to presurgical patients with hippocampal sclerosis (HS) mesial TLE, while the causes of TLE are more complex including focal cortical dysplasia (FCD), nonspecific gliosis, and dysembryoplastic neuroepithelial tumors (DNET) [39]. Moreover, the presurgical patients usually have intense structural and functional abnormalities, limiting the clinical applications of these methods. To ensure more general applications, the adopted TLE were not pre-selected in this study. Nevertheless, the accuracies of the proposed framework were similar to those of SVM-VBM approaches. Consequently, it can be assumed that our method may work well in TLEs with different etiological factors.

In conclusion, the proposed deep learning framework for ^18^F-FDG PET can accurately and efficiently localize epileptic foci and determine metabolic abnormality, which might be applied as a future computer-assisted diagnosis tool for epilepsy patients.

## Supplementary information

ESM 1 (DOCX 24 kb)
