# Supplementary material for: A deep learning framework for 18F-FDG PET imaging diagnosis in pediatric patients with temporal lobe epilepsy
Source: Eur J Nucl Med Mol Imaging. 2021 Jan 9;48(8):2476–85. doi: 10.1007/s00259-020-05108-y (PMC8241642; doi:10.1007/s00259-020-05108-y)
Supplement: Supplementary file 1 — (DOCX 24 kb) [file 259_2020_5108_MOESM1_ESM.docx]

Ms. No. EJNM-D-20-00913R1

**A Deep Learning Framework for ^18^F-FDG PET Imaging Diagnosis in Pediatric Patients with Temporal Lobe Epilepsy**

**Supplemental Materials**

**Materials and Methods**

**Radiomics-based ^18^F-FDG PET image analysis**

A total 386 features, including 344 multiscale wavelet features, 36 texture features and 6 intensity features, were shown in Table S1. The multiscale wavelet features included the textures and intensities of coif1 wavelet transformed images. The texture features were calculated based on histogram, gray level co-occurrence matrix (GLCM), neighborhood gray-tone difference matrix (NGTDM) and gray level zone size matrix (GLZSM). The intensity features included mean, variance, skewness, kurtosis, energy, entropy.

**Table S1** Summary of radiomic features for symmetricity analysis.

| **Feature Category**  **(count)** | **Specific Feature** | |
| --- | --- | --- |
| Multiscale wavelet features (344) | Mean of wavelet transform dec2* | |
|  | Mean of wavelet transform dec6* | |
|  | Variance of wavelet transform dec2* | |
|  | Variance of wavelet transform dec7* | |
|  | Contrast 1 of wavelet transform dec4* | |
|  | Contrast 1 of wavelet transform dec6* | |
|  | Dissimilarity of wavelet transform dec4* | |
|  | Sum of squares variance of wavelet transform dec2* | |
|  | and 336 other features | |
|  |  |  |
| Texture features (36) | High gray-level zone emphasis* | |
|  | Large zone / high gray emphasis* | |
|  | Angular second moment | |
|  | Contrast 1 | |
|  | Correlation | |
|  | Sum of squares variance | |
|  | Inverse difference moment | |
|  | Sum average | |
|  | Sum variance | |
|  | Sum entropy | |
|  | Entropy | |
|  | Difference variance | |
|  | Difference entropy | |
|  | Information correlation 1 | |
|  | Information correlation 2 | |
|  | Maximal correlation coefficient | |
|  | Autocorrelation | |
|  | Dissimilarity | |
|  | Cluster shade | |
|  | Cluster prominence | |
|  | Maximum probability | |
|  | Inverse difference | |
|  | Coarseness | |
|  | Contrast 2 | |
|  | Busyness | |
|  | Complexity | |
|  | Texture strength | |
|  | Small zone size emphasis | |
|  | Large zone size emphasis | |
|  | Low gray-level zone emphasis | |
|  | Small zone / low gray emphasis | |
|  | Small zone / high gray emphasis | |
|  | Large zone / low gray emphasis | |
|  | Gray-level non-uniformity | |
|  | Zone size non-uniformity | |
|  | Zone size percentage | |
|  | |  |
| Intensity features (6) | Mean | |
|  | Variance | |
|  | Skewness | |
|  | Kurtosis | |
|  | Energy | |
|  | Entropy | |

* Selected features in the radiomics model.
